# Comparison of concomitant injuries and patient-reported outcome in patients that have undergone both primary and revision ACL reconstruction—a national registry study

**DOI:** 10.1186/s13018-019-1532-z

**Published:** 2020-01-10

**Authors:** Eleonor Svantesson, Eric Hamrin Senorski, Frida Kristiansson, Eduard Alentorn-Geli, Olof Westin, Kristian Samuelsson

**Affiliations:** 1 Department of Orthopedics, Institute of Clinical Sciences, The Sahlgrenska Academy, University of Gothenburg, Gothenburg, Sweden; 2 Department of Health and Rehabilitation, Institute of Neuroscience and Physiology, The Sahlgrenska Academy, University of Gothenburg, Gothenburg, Sweden; 3 Fundación García Cugat, Barcelona, Spain; 4 Artroscopia GC, Hospital Quirón, Barcelona, Spain; 5 Mutualidad Catalana de Futbolistas, Federación Catalana de Fútbol, Barcelona, Spain; 6 Department of Orthopedics, Sahlgrenska University Hospital, Mölndal, Sweden

**Keywords:** ACL, Anterior cruciate ligament, Reconstruction, Revision, Registry, Outcome, Autograft, Meniscus, Cartilage

## Abstract

**Background:**

Anterior cruciate ligament (ACL) revision surgery has been associated with inferior outcome compared with primary ACL reconstruction. However, this has rarely been investigated in a consecutive cohort limited to patients that have undergone both primary and revision ACL reconstruction. This study aimed to assess differences in outcome and concomitant injuries between primary and revision ACL reconstruction in such a cohort, and to identify predictors of the patient-reported outcome after ACL revision.

**Methods:**

Patients who had undergone both primary and revision ACL reconstruction were identified in the Swedish National Knee Ligament Registry. Patients aged 13–49 years with hamstring tendon primary ACL reconstruction and data on the Knee Injury and Osteoarthritis Outcome Score (KOOS) on at least one occasion (preoperative or one year postoperatively) at both surgeries were eligible. Concomitant injuries and the KOOS were compared between each patient’s primary and revision ACL reconstruction. Linear regression analyses were performed to determine predictors of the one-year KOOS after ACL revision.

**Results:**

A total of 1014 patients were included. Cartilage injuries increased at ACL revision (*p* < 0.001), as 23.0% had a cartilage injury at ACL revision that was not present at primary ACL reconstruction. The 1-year KOOS was lower after ACL revision compared with primary ACL reconstruction, with the largest difference in the KOOS sports and recreation (5.2 points, SD 32.2, *p* = 0.002). A posterolateral corner (PLC) injury at ACL revision was a negative predictor of KOOS, with the largest effect on the sports and recreation subscale (β = − 29.20 [95% CI − 50.71; − 6.69], *p* = 0.011). The use of allograft for ACL revision was an independent predictor of a poorer KOOS QoL (β = − 12.69 [95% CI − 21.84; − 3.55], *p* = 0.0066) and KOOS_4_ (β = − 11.40 [95% CI − 19.24; − 3.57], *p* = 0.0044).

**Conclusion:**

Patients undergoing ACL revision reported a 1-year outcome that was slightly inferior to the 1-year outcome after their primary ACL reconstruction. An ACL revision was associated with an increase in cartilage injuries. A PLC injury at ACL revision and the use of allograft for ACL revision predicted a clinically relevant poorer KOOS one year after ACL revision.

## Background

Despite the increased knowledge and evolution in reconstructive surgery of the anterior cruciate ligament (ACL) over the past few decades, graft failures and residual knee laxity remain a concern. Data from large knee ligament registries have shown that the probability of needing ACL revision surgery within 3 years of the primary ACL reconstruction is generally low, ranging from 2.8 to 3.7% [1]. Within 10 years, however, it is estimated that at least one in nine patients may opt for an ACL revision due to re-rupture or signs of clinical failure [2].

An ACL revision has been associated with inferior patient- and clinician-reported outcome and a greater likelihood of developing tibiofemoral osteoarthritis compared with a primary ACL reconstruction [3, 4]. It has also been shown that the prevalence of concomitant intra-articular injuries among ACL revisions, in particular the prevalence of cartilage injuries, is high [5–8]. Moreover, the failure rate for ACL revisions is nearly three to four times higher compared with primary ACL reconstructions [4].

Although previous registry-based studies have unanimously concluded that the patient-reported outcome after ACL revision compared with primary ACL reconstruction is inferior [8–11], comparisons of the patient-reported outcome in a consecutive cohort limited to patient that have undergone both primary and revision ACL reconstruction are lacking. Additionally, an analysis of the same cohort at primary and revision ACL reconstruction would make it possible to evaluate the course of concomitant injuries identified at the primary ACL reconstruction in each individual, and how they affect the outcome of the ACL revision. An in-depth understanding of the prognosis of an ACL revision could aid both clinicians and patients in deciding whether an ACL revision is indicated and how to optimize the outcome with reasonable expectations.

The purpose of this study was therefore to compare the patient-reported outcome and prevalence of concomitant knee injuries in a consecutive cohort that had undergone both primary and revision ACL reconstruction. This study also sought to identify patient-, injury-, and surgery-related factors predictive of the patient-reported outcome 1 year after ACL revision. It was hypothesized that ACL revision would be associated with an inferior patient-reported outcome, as well as an increase in the prevalence of concomitant injuries, compared with primary ACL reconstruction.

## Methods

### Study population

Patients registered in the Swedish National Knee Ligament Registry (SNKLR) that had undergone ACL revision and also had a registered primary ACL were assessed for eligibility. Patients aged 13 to 49 years who received hamstring tendon (HT) autografts at the primary ACL reconstruction were eligible for inclusion. Hamstring tendon autografts are used in over 90% of all primary ACL reconstructions in Sweden [12], and another reason for limiting inclusion to HT autograft at primary ACL reconstruction was to obtain a homogenous study population with regard to graft choice at baseline. The graft choice at ACL revision was however not considered in the eligibility process as it was part of the study purpose to assess whether graft choice at ACL revision affected the outcome. Additionally, included patients needed to have available data for the Knee Injury and Osteoarthritis Outcome Score (KOOS) on at least one occasion (preoperative or 1 year) at both the primary and revision ACL reconstruction. Patients were excluded if they had a contralateral ACL reconstruction registered or underwent a contralateral ACL reconstruction within 2 years of the ACL revision since bilateral ACL reconstruction previously has been shown to result in inferior KOOS compared with unilateral ACL reconstruction [8] and, thus, could have affected the analysis. Finally, patients who had sustained a concomitant fracture, nerve, or vascular injury at either ACL reconstruction were excluded.

### The Swedish National Knee Ligament Registry

The SNKLR was established in 2005 and serves as a nationwide database with high coverage and compliance, including more than 90% of all ACL reconstructions performed annually in Sweden [13]. The surgeon-reported section includes reports of patient-, injury-, and surgery-related factors. All surgical procedures and intra-operative findings are documented. If a patient requires additional surgery, such as an ACL revision, this is registered as a separate entry which is linked to the patient’s primary surgery. The patient-reported part includes prospectively collected data in terms of the European Quality of Life-5 Dimensions (EQ-5D) and the KOOS, which are assessed at standardized timepoints following each ACL reconstruction. A detailed description of data collection has previously been reported [9].

### Variables

Variables related to the following categories were extracted for the primary and revision ACL reconstruction: patient demographics, surgery-related factors, and intra-operatively identified concomitant injuries. Patient demographics included patient sex, age, and activity that led to injury/revision. The activity variable in the SNKLR includes sport and work-related activities, as well as activities of daily living (ADL). For this study, the most frequently reported sporting activities were reported separately, and the remainder were categorized as “other.” For surgery-related factors, the time from primary ACL reconstruction to ACL revision, graft type, and graft fixation were reported. The presence of intra-operatively identified concomitant injuries included meniscal, cartilage, and other ligament injuries. Injuries to the menisci were separated for medial and lateral injury and data on whether surgical treatment was performed (resection or repair) were extracted. The presence of cartilage injury was reported as yes or no and was further assessed for location (patella, femoral condyles, tibial plateaus, and trochlea) and severity according to the International Cartilage Repair Society (ICRS). The ICRS grading is based on cartilage lesion depth and ranges from 0 to 4. An ICRS of 0 represents normal cartilage, ICRS 1-2 involve less than 50% of the cartilage thickness, and ICRS 3-4 involve more than 50% of the cartilage thickness [14]. Concomitant injuries to other ligaments included the surgeon reporting a presence (Yes/No) of medial or lateral collateral ligament injury (MCL or LCL), posterior cruciate ligament (PCL) injury, and posterolateral corner (PLC) injury at time of ACL reconstruction.

### Outcome measurement

The patient-reported outcome measurement used was the KOOS. The KOOS comprises five subscales—pain, knee-related symptoms, ADL, function in sports and recreation, and knee-related quality of life (QoL). The maximum score on each subscale is 100, which indicates no knee problems, while a score of zero represents the worst possible state [15]. The KOOS_4_ is an average score (ranging between 0 and 100) of four subscales, where the ADL subscale has been excluded [16]. The KOOS_4_ has been developed as a modification of the original KOOS to avoid a ceiling effect, since an ACL injury rarely causes problems in the ADL. For the purpose of this study, separate analyses were carried out for the KOOS_4_ and each KOOS subscale, preoperatively and 1 year after primary and revision ACL reconstruction. The KOOS_4_, KOOS sports and recreation, and KOOS QoL were regarded as the main outcomes in the predictive analysis of one-year outcome post-ACL revision. This was chosen since the KOOS sports and recreation subscale and the QoL subscales have been shown to be the most responsive subscales [17].

### Statistical analysis

Statistical analyses were performed using the SAS statistical analysis system (SAS/STAT, version 14.2, 2016; SAS Institute Inc., Cary, North Carolina, USA). Descriptive statistics were reported as the mean and standard deviation (SD) and the median and range for continuous variables and as the count and proportion for categorical variables. The sign test was applied to analyze the change in the prevalence, treatment, and severity of concomitant injuries from primary to revision ACL reconstruction. Specifically, the sign test was used to analyze the difference in the proportion of patients with an increase or a decrease for each investigated concomitant injury from primary to revision ACL reconstruction. For injuries and performance of surgical treatments that were reported as “yes” or “no,” a decrease was defined as a change from “yes” at the primary ACL reconstruction to “no” at the ACL revision and an increase was defined as a change from “no” to “yes.” The severity of cartilage injuries ranged from “no injury or ICRS 0” to ICRS 4 and a decrease or increase referred to a patient changing one or more grades in either direction from primary to revision ACL reconstruction. The KOOS subscales and the KOOS_4_ were analyzed as continuous variables and the preoperative and 1-year KOOS for primary and revision ACL reconstruction were compared over time using Wilcoxon signed rank test. In order to find predictors of the 1-year outcome after ACL revision, univariable linear regression models were used with the KOOS sports and recreation, KOOS QoL, and KOOS_4_ as dependent variables in separate analyses. The distribution of the residuals of the dependent variables was inspected in a histogram and fulfilled the assumption of normal distribution. Independent variables for the analyses and the way specific variables were analyzed are presented in Table 1. Subsequently, a forward stepwise multivariable linear regression was performed. Predictors that reached a *p* value of < 0.10 in the univariable analysis were entered in the forward stepwise multivariable regression model. The results of the regression models were presented with β-coefficients, 95% confidence intervals (CI), and *p* values. All significance tests were two-sided and conducted at the 5% significance level.

**Table 1:** Variables analyzed as predictors of the one-year knee injury and osteoarthritis outcome score after anterior cruciate ligament revision

Variable category	Variable
Patient demographics	• Age^a^ (continuous variable per 10 years)
	• Patient sex (male/female)
Surgery related	• Years from primary ACL reconstruction to ACL revision (continuous variable per year)
	• Graft type at ACL revision (hamstring tendon, patellar tendon, quadriceps tendon, allograft, other)
Concomitant injuries	
Meniscal injuries	• Any meniscal injury (yes/no)^a^
	• Medial meniscus injury (yes/no)^a^
	• Lateral meniscus injury (yes/no)^a^
	• Surgical treatment of any meniscal injury (yes/no)^a^
	• Meniscal resection of any meniscal injury (yes/no)^a^
	• Meniscal repair of any meniscal injury (yes/no)^a^
Cartilage injuries	• Any cartilage injury (yes/no)^a^
	• Highest grade on any cartilage injury (ordinal variable – ICRS 0/ICRS 1-2/ICRS 3-4)^a^
	• Patella (dichotomized ICRS 0-2/ICRS 3-4)^a^
	• Tibial plateaus (dichotomized ICRS 0-2/ICRS 3-4)^a^
	• Femoral condyles (dichotomized ICRS 0-2/ICRS 3-4)^a^
	• Trochlea (dichotomized ICRS 0-2/ICRS 3-4)^a^
Ligament injuries	• MCL (yes/no)^a^
	• LCL (yes/no)^a^
	• PCL (yes/no)^a^
	• PLC (yes/no)^a^

*ACL* anterior cruciate ligament, *ICRS* International Cartilage Repair Society, *LCL* lateral collateral ligament, *MCL* medial collateral ligament, *PCL* posterior cruciate ligament, *PLC* posterolateral corner

^a^The status of the variable at both primary and revision ACL reconstruction was analyzed. The information in parentheses indicates how the variable was analyzed

## Results

**Fig. 1:**
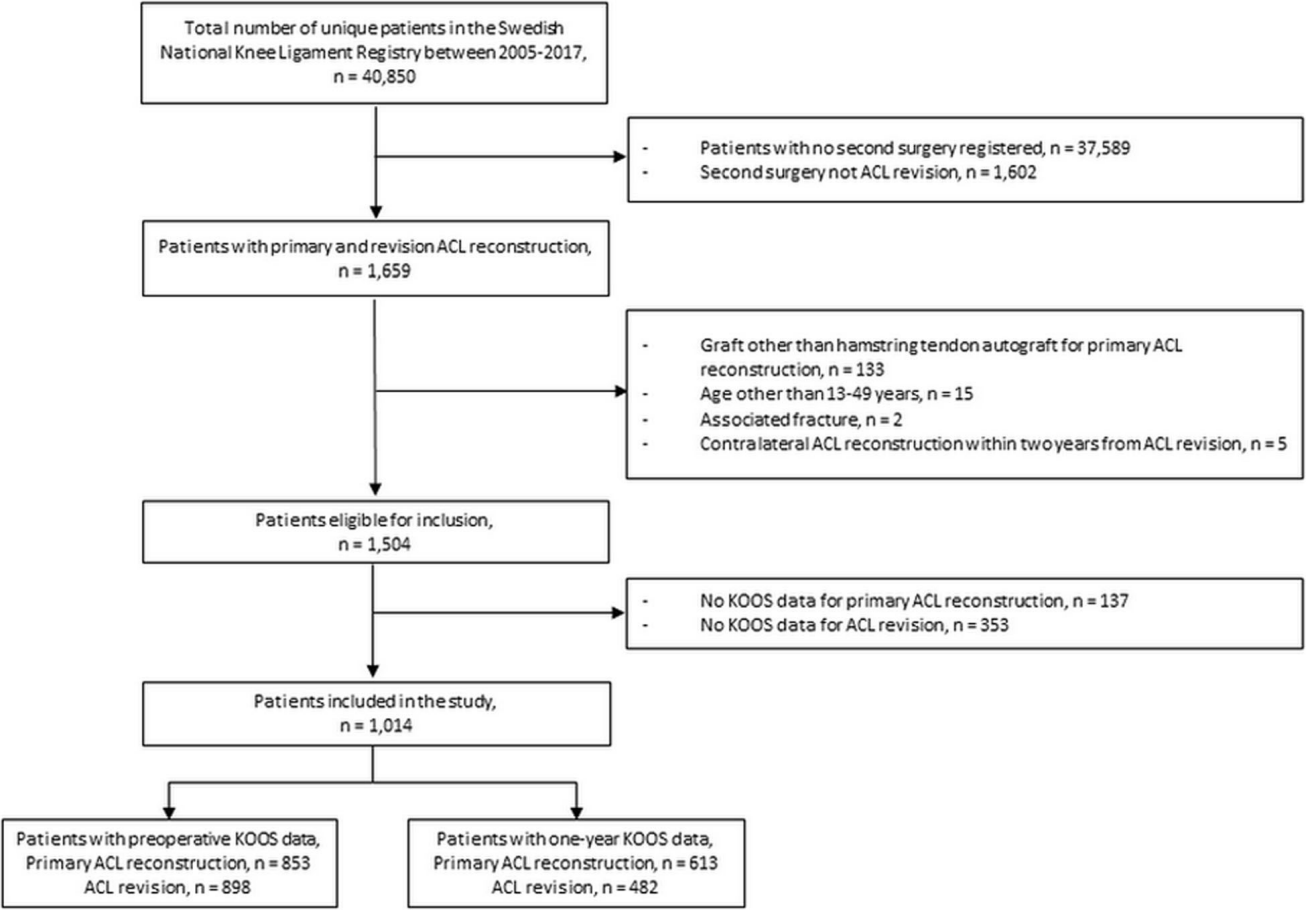
Flow chart of patient inclusion

A total of 40,850 patients were assessed for eligibility in the SNKLR, of which 1014 patients met the inclusion criteria (Fig. 1). The study cohort consisted of 51.3% men and the mean age was 21.5 years at primary ACL reconstruction and 24.2 years at ACL revision. The study demographic data is presented in Table 2.

**Table 2:** Patient demographics and surgical characteristics for primary and revision ACL reconstruction

	Primary ACL reconstruction *n* = 1014	ACL revision *n* = 1014
Patient sex		
Female	494 (48.7%)	494 (48.7%)
Male	520 (51.3%)	520 (51.3%)
Age at ACL reconstruction	21.5 (7.2) 19.0 (13.0; 49.0)	24.2 (7.5) 22.0 (14.0; 53.0)
Time from primary ACL reconstruction to ACL revision (years)		2.7 (2.0) 2.0 (0.2; 12.3)
Activity at time of injury		
Soccer	520 (51.4%)	412 (40.9%)
Floorball	89 (8.8%)	57 (5.7%)
Handball	76 (7.5%)	58 (5.8%)
Alpine	83 (8.2%)	47 (4.7%)
Other	244 (24.1%)	434 (43.1%)
Missing	2	6
ACL graft choice		
Patellar tendon autograft		639 (64.4%)
Hamstring tendon autograft	1014 (100.0%)	190 (19.1%)
Quadriceps tendon autograft		83 (8.4%)
Allograft		69 (6.9%)
Other		12 (1.2%)
Missing	0	21
Tibial fixation		
Metal screw	266 (26.5%)	553 (55.5%)
Resorbable screw	182 (18.1%)	171 (17.2%)
AO screw	226 (22.5%)	42 (4.2%)
Retro screw	39 (3.9%)	18 (1.8%)
Intrafix/Rigidfix	109 (10.9%)	4 (0.4%)
Endo-/Retrobutton/Tightrope	52 (5.2%)	75 (7.5%)
Metal screw + staple/osteosuture	109 (10.9%)	92 (9.2%)
Other	21 (2.1%)	41 (4.1%)
Missing	10	18
Femoral fixation		
Endo-/Retrobutton/Tightrope	645 (64.2%)	458 (46.1%)
Rigidfix/Transfix	193 (19.2%)	8 (0.8%)
Metal screw	150 (14.9%)	509 (51.2%)
Other	17 (1.7%)	19 (1.9%)
Missing	9	20

For categorical variables, *n* (%) is presented

For continuous variables, the mean (SD)/median (min; max)/*n* = is presented. *ACL* anterior cruciate ligament, *n* number

### Concomitant knee joint injuries

Concomitant knee joint injuries at primary and revision ACL reconstruction are presented in Table 3. Meniscal injuries decreased at ACL revision (*p* < 0.001), as the reported meniscal status changed from injured at primary ACL reconstruction to uninjured at ACL revision in 277 patients (27.3%), while 183 patients (18.0%) had their first registered meniscal injury at ACL revision. There was no change in the number of medial meniscal injuries between primary and revision ACL reconstruction, while the lateral meniscal injuries decreased (*p* < 0.001). The prevalence of cartilage injuries increased from primary to revision ACL reconstruction (*p* < 0.001), as 23.0% of the cohort were identified with a cartilage injury at ACL revision that was not present at primary ACL reconstruction. There was a decrease in reported MCL injuries at ACL revision compared with primary ACL reconstruction (3.3% versus 1.2%, *p* = 0.003). Concomitant PLC injuries increased significantly between the surgeries (*p* = 0.002) and were present in three patients (0.3%) at primary ACL reconstruction compared with 16 patients (1.6%) at ACL revision.

**Table 3:** Comparison of concomitant injuries at primary and revision anterior cruciate ligament reconstruction

	Primary ACL reconstruction (*n* = 1014)	ACL revision (*n* = 1014)	Change from primary ACL reconstruction to revision	*p* value^a^
Any meniscal injury (MM and/or LM) Yes	436 (43.0%)	342 (33.7%)	Decrease 277 (27.3%) Equal 554 (54.6%) Increase 183 (18.0%)	< 0.001
MM injury Yes	223 (22.0%)	209 (20.6%)	Decrease 164 (16.2%) Equal 700 (69.0%) Increase 150 (14.8%)	0.46
LM injury Yes	282 (27.8%)	192 (18.9%)	Decrease 211 (20.8%) Equal 682 (67.3%) Increase 121 (11.9%)	< 0.001
Meniscal surgical treatment Yes	372 (36.7%)	314 (31.0%)	Decrease 248 (24.5%) Equal 576 (56.8%) Increase 190 (18.7%)	0.006
MM resection Yes	149 (14.7%)	127 (12.5%)	Decrease 128 (12.6%) Equal 780 (76.9%) Increase 106 (10.5%)	0.17
MM repair Yes	41 (4.0%)	63 (6.2%)	Decrease 37 (3.6%) Equal 918 (90.5%) Increase 59 (5.8%)	0.032
LM resection Yes	180 (17.8%)	131 (12.9%)	Decrease 154 (15.2%) Equal 755 (74.5%) Increase 105 (10.4%)	0.003
LM repair Yes	45 (4.4%)	45 (4.4%)	Decrease 40 (3.9%) Equal 934 (92.1%) Increase 40 (3.9%)	1.00
Cartilage injury Yes	186 (18.3%)	356 (35.1%)	Decrease 63 (6.2%) Equal 718 (70.8%) Increase 233 (23.0%)	< 0.001
MCL injury Yes	37 (3.6%)	16 (1.6%)	Decrease 33 (3.3%) Equal 969 (95.6%) Increase 12 (1.2%)	0.003
LCL injury Yes	6 (0.6%)	12 (1.2%)	Decrease 5 (0.5%) Equal 998 (98.4%) Increase 11 (1.1%)	0.21
PCL injury Yes	8 (0.8%)	8 (0.8%)	Decrease 5 (0.5%) Equal 1004 (99.0%) Increase 5 (0.5%)	1.00
PLC injury Yes	3 (0.3%)	16 (1.6%)	Decrease 2 (0.2%) Equal 997 (98.3%) Increase 15 (1.5%)	0.002

For the comparison of decrease versus increase in each concomitant injury, Yes indicates the presence of the described concomitant injury

*ACL* anterior cruciate ligament, *MCL* medial collateral ligament, *MM* medial meniscus, *LCL* lateral collateral ligament, *LM* lateral meniscus, *PCL* posterior cruciate ligament, *PLC* posterolateral corner

Table 4 presents the ICRS grades for and locations of cartilage injuries and comparisons between primary and revision ACL reconstruction. A total of 263 patients (26.1%) had an increase in the reported ICRS grade at ACL revision compared with primary ACL reconstruction, while 75 patients (7.4%) had an improvement in ICRS grade (*p* < 0.001). At ACL revision, 93 patients (9.2%) presented with a grade 3–4 cartilage injury compared with 35 patients (3.5%) at the primary ACL reconstruction. The most common location of cartilage injuries was the femoral condyles, where 231 patients (22.9%) had a worsening in ICSR grade between primary and ACL revision and 77 patients (7.6%) had an improvement in ICRS grade (*p* < 0.001).

**Table 4:** Location and severity of concomitant cartilage injuries at primary and revision anterior cruciate ligament reconstruction

	Primary ACL reconstruction (*n* = 1014)^a^	ACL revision (*n* = 1014)^b^	Change from primary ACL reconstruction to revision	*p* value^c^
Highest ICRS on any location				
No injury	828 (81.7%)	658 (65.1%)	Decrease 75 (7.4%) Equal 671 (66.5%) Increase 263 (26.1%)	< 0.001
ICRS 1-2	150 (14.8%)	259 (25.6%)		
ICRS 3-4	35 (3.5%)	93 (9.2%)		
Patella				
No injury	984 (97.1%)	941 (93.2%)		< 0.001
ICRS 1	14 (1.4%)	30 (3.0%)	Decrease 18 (1.8%) Equal 931 (92.3%) Increase 60 (5.9%)	
ICRS 2	14 (1.4%)	28 (2.8%)		
ICRS 3	1 (0.1%)	10 (1.0%)		
ICRS 4	0 (0.0%)	1 (0.1%)		
Tibial plateaus				
No injury	961 (94.9%)	894 (88.5%)		< 0.001
ICRS 1	21 (2.1%)	50 (5.0%)	Decrease 30 (3.0%) Equal 880 (87.2%) Increase 99 (9.8%)	
ICRS 2	27 (2.7%)	50 (5.0%)		
ICRS 3	4 (0.4%)	12 (1.2%)		
ICRS 4	0 (0.0%)	4 (0.4%)		
Femoral condyles				
No injury	859 (84.8%)	719 (71.2%)		< 0.001
ICRS 1	49 (4.8%)	96 (9.5%)	Decrease 77 (7.6%) Equal 701 (69.5%) Increase 231 (22.9%)	
ICRS 2	74 (7.3%)	121 (12.0%)		
ICRS 3	21 (2.1%)	54 (5.3%)		
ICRS 4	10 (1.0%)	20 (2.0%)		
Trochlea				
No injury	1002 (98.9%)	960 (95.0%)		< 0.001
ICRS 1	7 (0.7%)	17 (1.7%)	Decrease 6 (0.6%) Equal 954 (94.5%) Increase 49 (4.9%)	
ICRS 2	4 (0.4%)	19 (1.9%)		
ICRS 3	0 (0.0%)	7 (0.7%)		
ICRS 4	0 (0.0%)	7 (0.7%)		

*ACL* anterior cruciate ligament, *ICRS* international cartilage repair score

^a^Missing data on ICRS for one patient

^b^Missing data on ICRS for four patients

^c^For the comparison of decrease versus increase in cartilage injury severity

### The Knee Injury and Osteoarthritis Outcome Score

The comparisons of the KOOS are presented in Table 5. The preoperative KOOS for the ACL revision was significantly higher across all the subscales except the ADL and QoL, compared with the preoperative KOOS at primary ACL reconstruction. Conversely, the 1-year postoperative KOOS was significantly inferior after the ACL revision compared with the primary ACL reconstruction on all subscales except the QoL. The largest difference in the mean 1-year KOOS between primary and revision ACL reconstruction was in the sports and recreation subscale (5.2 points, SD 32.2, *p* = 0.002), followed by the pain subscale (3.1 points, SD 20.1, *p* = 0.003).

**Table 5:** The Knee injury and Osteoarthritis Outcome Score for primary and revision ACL reconstruction

	Primary ACL reconstruction (*n* = 1014)	ACL revision (*n* = 1014)	Change from primary ACL reconstruction to revision	*p* value
Preoperative KOOS				
Pain	75.2 (17.9) 77.8 (0.0; 100.0) *n* = 853	76.1 (19.2) 80.6 (11.1; 100.0) *n* = 898	1.3 (18.8) 0.0 (− 83.3; 66.7) *n* = 754	0.017
Symptoms	69.7 (18.3) 71.4 (3.6; 100.0) *n* = 852	70.9 (19.3) 71.4 (10.7; 100.0) *n* = 898	1.7 (20.7) 0.0 (− 75.0; 67.9) *n* = 753	0.032
ADL	84.9 (16.7) 91.2 (0.0; 100.0) *n* = 853	85.0 (17.5) 92.7 (2.9; 100.0) *n* = 897	0.2 (17.5) 0.000 (− 94.1; 61.8) *n* = 753	0.37
Sports and recreation	42.2 (28.0) 40.0 (0.0; 100.0) *n* = 853	45.4 (29.9) 45.0 (0.0; 100.0) *n* = 898	3.7 (30.7) 0.0 (− 75.0; 100.0) *n* = 754	0.002
QoL	34.3 (19.7) 31.3 (0.0; 100.0) *n* = 853	34.9 (24.0) 31.3 (0.0; 100.0) *n* = 898	1.4 (25.0) 0.0 (− 81.3; 100.0) *n* = 754	0.86
KOOS_4_	55.3 (17.7) 55.7 (0.9; 100.0) *n* = 853	56.8 (20.4) 56.5 (7.9; 100.0) *n* = 898	2.0 (19.7) 1.9 (− 59.4; 61.5) *n* = 754	0.012
One−year postoperative KOOS				
Pain	79.9 (18.7) 86.1 (5.6; 100.0) *n* = 613	78.2 (20.6) 84.7 (5.6; 100.0) *n* = 482	− 3.1 (20.1) − 2.8 (− 63.9; 91.7) *n* = 303	0.003
Symptoms	72.8 (19.6) 75.0 (14.3; 100.0) *n* = 612	71.1 (21.1) 75.0 (3.6; 100.0) *n* = 482	− 2.8 (20.1) 0.0 (− 64.3; 60.7) *n* = 302	0.014
ADL	88.0 (16.3) 94.1 (11.8; 100.0) *n* = 612	86.6 (18.3) 94.1 (2.9; 100.0) *n* = 482	− 2.6 (16.1) 0.0 (− 61.8; 57.4) *n* = 302	0.006
Sports and recreation	56.0 (31.0) 60.0 (0.0; 100.0) *n* = 613	51.9 (30.3) 52.5 (0.0; 100.0) *n* = 482	− 5.2 (32.2) − 5.0 (− 95.0; 100.0) *n* = 303	0.002
QoL	46.8 (28.2) 43.8 (0.0; 100.0) *n* = 613	45.8 (26.2) 43.8 (0.0; 100.0) *n* = 482	− 1.8 (29.3) 0.0 (− 81.3; 93.8) *n* = 303	0.17
KOOS_4_	63.8 (22.2) 66.9 (7.9; 100.0) *n* = 613	61.7 (22.4) 64.6 (3.7; 100.0) *n* = 482	− 3.2 (22.3) − 3.2 (− 59.8; 62.3) *n* = 303	0.01
KOOS change preoperative to one−year follow up				
Pain	5.8 (17.6) 5.6 (− 55.6; 66.7) *n* = 452	2.8 (19.7) 2.8 (− 75.0; 72.2) *n* = 366	− 3.2 (28.2) − 2.8 (− 77.8; 94.5) *n* = 201	0.033
Symptoms	4.3 (19.6) 3.6 (− 78.6; 60.7) *n* = 451	0.6 (20.6) 0.0 (− 89.3; 89.3) *n* = 366	− 4.5 (.)(− 82.1; 121.4) *n* = 200	0.011
ADL	3.7 (15.9) 1.5 (− 55.9; 61.8) *n* = 451	2.1 (15.6) 0.0 (− 54.4; 55.9) *n* = 365	− 0.6 (22.7) − 1.5 (− 57.4; 97.1) *n* = 200	0.088
Sports and recreation	16.4 (31.3) 15.0 (− 85.0; 100.0) *n* = 452	8.1 (32.7) 5.0 (− 100.0; 100.0) *n* = 366	− 7.1 (46.9) − 10.0 (− 115.0; 130.0) *n* = 201	0.024
QoL	15.6 (28.4) 12.5 (− 100.0; 87.5) *n* = 452	12.1 (29.9) 12.5 (− 100.0; 93.8) *n* = 366	− 3.8 (44.5) − 6.3 (− 137.5; 125.0) *n* = 201	0.26
KOOS_4_	10.5 (20.6) 11.3 (− 71.8; 69.9) *n* = 452	5.9 (22.5) 5.9 (− 91.1; 71.4) *n* = 366	− 4.6 (32.00) − 4.2 (− 79.2; 99.3) *n* = 201	0.028

The mean (SD)/median (min; max)/*n* = is presented

For comparison over time, the Wilcoxon signed rank test was used

*KOOS* Knee injury and Osteoarthritis Outcome Score, *ADL* activities of daily living, *QoL* quality of life

A significantly larger improvement from the preoperative KOOS to the 1-year KOOS was found with the primary ACL reconstruction compared with the ACL revision in the subscales of pain, symptoms and sports and recreation. The improvement in the sports and recreation subscale was 7.1 points (SD 46.9) lower with the ACL revision compared with the primary ACL reconstruction (*p* = 0.024). The difference in the symptoms subscale was 4.5 points (*p* = 0.011) and in the pain subscale 3.2 points (SD 28.2, *p* = 0.033).

### Predictors of 1-year outcome after ACL revision

A total of 482 patients had reported data for the 1-year follow-up after ACL revision and were included in the predictive analysis of the 1-year KOOS after ACL revision. Higher age at primary ACL reconstruction predicted a poorer outcome for KOOS sports and recreation and KOOS_4_ in the univariable analysis (per 10-year increment in age). No other variable from the primary ACL reconstruction was a significant predictor of the one-year KOOS after ACL revision (data presented in Appendix).

**Fig. 2:**
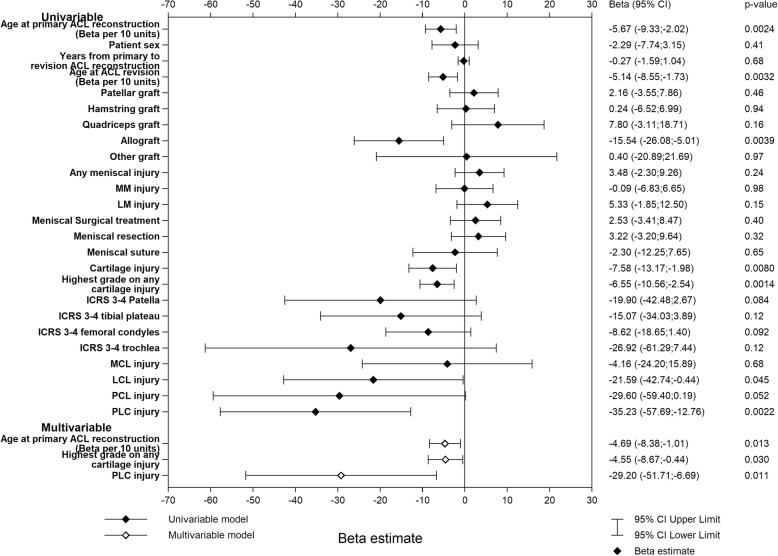
Univariable and multivariable linear regression models of KOOS sports and recreation one year after ACL revision. Graft type is the graft used for ACL revision. Concomitant injuries refer to the status at the time of ACL revision. *ACL* anterior cruciate ligament, *ICRS* international cartilage repair score, *MCL* medial collateral ligament, *MM* medial meniscus, *LCL* lateral collateral ligament, *LM* lateral meniscus, *PCL* posterior cruciate ligament, *PLC* posterolateral corner

With regard to the sports and recreation subscale, the following factors at ACL revision were significant negative predictors in the univariable analysis (*p* < 0.05); higher age, the use of allograft, a concomitant cartilage injury, a higher ICRS grade of cartilage injury (any location), an LCL injury, and a PLC injury, with β values ranging from − 35.23 (95% CI − 57.69; − 12.76) for a PLC injury to − 5.14 (95% CI − 8.55; − 1.73) for every 10-year increase in age. In the multivariable model, significant predictors were higher ICRS of any cartilage injury (β = − 4.55 [95% CI − 8.67; − 0.44], *p* = 0.030) and the presence of a PLC injury (β = − 29.20 [95% CI − 50.71; − 6.69], *p* = 0.011) at the time of ACL revision. Specifically, every one-step increment in ICRS category (ICRS 0, ICRS 1-2, ICRS 3-4) predicted 4.55 points poorer KOOS sports and recreation. Additionally, higher age at primary ACL reconstruction was a significant predictor of a poorer outcome in the multivariable model, resulting in a 4.69-point lower score for every 10-year increment in age (β = − 4.69 [95% CI − 8.38; − 1.01], *p* = 0.013). The multivariable model had an adjusted *R*^2^ of 0.041 (Fig. 2).

**Fig. 3:**
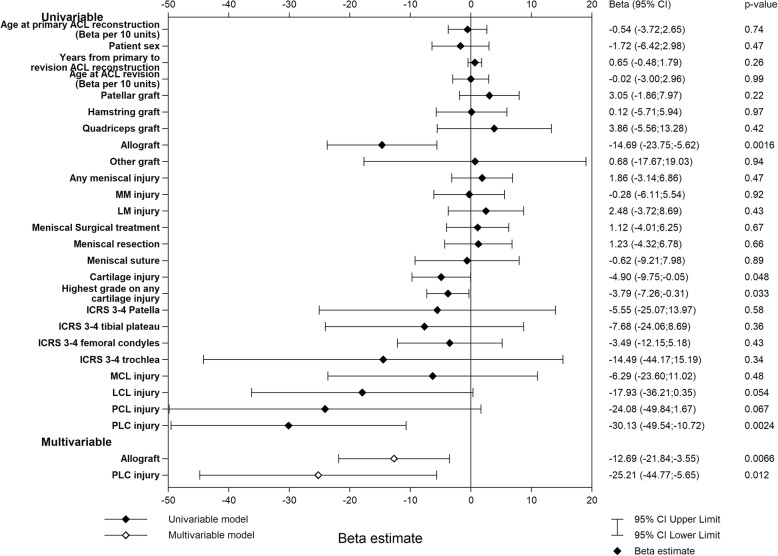
Univariable and multivariable linear regression models of KOOS quality of life one year after ACL revision. Graft type is the graft used for ACL revision. Concomitant injuries refer to the status at the time of ACL revision. *ACL* anterior cruciate ligament, *ICRS* international cartilage repair score, *MCL* medial collateral ligament, *MM* medial meniscus, *LCL* lateral collateral ligament, *LM* lateral meniscus, *PCL* posterior cruciate ligament, *PLC* posterolateral corner

For the QoL subscale, significant negative predictors at the time of ACL revision in the univariable analysis were the use of allograft, a concomitant cartilage injury, a higher ICRS grade of cartilage injury (any location), and a PLC injury, with β values ranging from − 30.13 (95% CI − 49.54; − 10.72) for a PLC injury to − 3.79 (95% CI − 7.26; − 0.31) for a one-unit increment in ICRS grade. Of these, the use of allograft (β = − 12.69 [95% CI − 21.84; − 3.55], *p* = 0.0066) and a concomitant PLC injury (β = − 25.21 [95% CI − 44.77; − 5.65], *p* = 0.012) were found to predict poorer QoL in the multivariable analysis (adjusted *R*^2^ = 0.030) (Fig. 3).

**Fig. 4:**
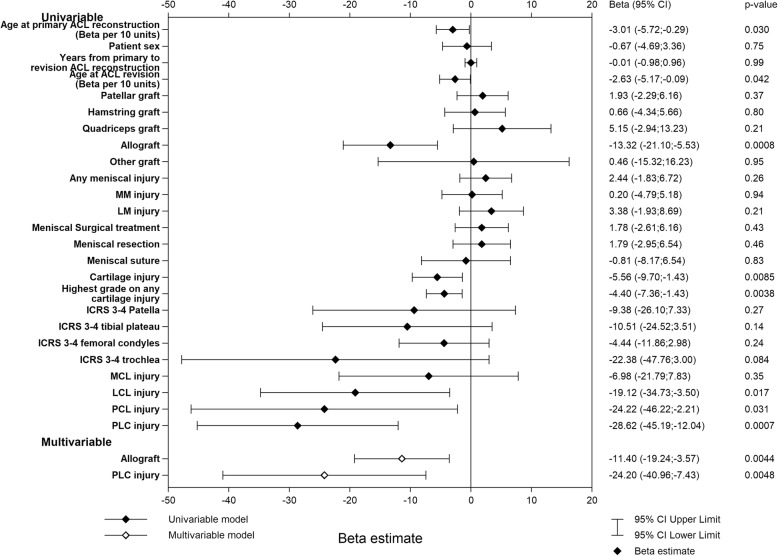
Univariable and multivariable linear regression models of the KOOS_4_ 1 year after ACL revision. Graft type is the graft used for ACL revision. Concomitant injuries refer to the status at the time of ACL revision. *ACL* anterior cruciate ligament, *ICRS* international cartilage repair score, *MCL* medial collateral ligament, *MM* medial meniscus, *LCL* lateral collateral ligament, *LM* lateral meniscus, *PCL* posterior cruciate ligament, *PLC* posterolateral corner

The univariable analysis of the KOOS_4_ identified the following negative predictors at the time of ACL revision; higher age, the use of allograft, a concomitant cartilage injury, a higher ICRS grade of cartilage injury (any location), an LCL injury, a PCL injury, and a PLC injury, with β values ranging from − 28.62 (95% CI − 45.19; − 12.04) for a PLC injury to − 2.63 (95% CI − 5.17; − 0.09) for higher age per 10 years. Significant predictors in the multivariable model were the use of allograft (β = − 11.40 [95% CI − 19.24; − 3.57], *p* = 0.0044) and a PLC injury (β = − 24.20 [95% CI − 40.96; − 7.43], *p* = 0.0048). The multivariable model had an adjusted *R*^2^ of 0.036 (Fig. 4).

Treatment for cartilage injuries was not analyzed as a predictor of outcome since the majority of the cartilage injuries were left in situ (data not shown).

## Discussion

This study showed that patients undergoing ACL revision reported a slightly inferior 1-year KOOS compared with their primary ACL reconstruction; however, the difference between the surgeries did not exceed the minimal important change in KOOS [17]. Nonetheless, an ACL revision was associated with a significant increase in concomitant injuries, especially in terms of cartilage injuries. More than one in four patients who have no concomitant cartilage injury at primary ACL reconstruction can be expected to present with one at ACL revision. The patient-, injury-, and surgery-related factors assessed at ACL revision had poor predictive ability and did not explain more than 5% of the variance in the one-year KOOS after the ACL revision. However, the strongest negative predictor of the 1-year KOOS after ACL revision was a concomitant PLC injury at ACL revision, which was an independent predictor of an almost 30-point poorer KOOS sports and recreation.

Several previous studies have concluded that the patient-reported outcome for patients undergoing ACL revision is poorer, when compared with an independent group of patients who have only undergone a primary ACL reconstruction [3, 4, 8–11]. However, patients who do not require an ACL revision may report a higher KOOS after the primary ACL reconstruction compared with those who subsequently require a revision [18], which could lead to an overestimation of the difference between primary and revision ACL reconstruction with regard to the perspective of an ACL revision patient. For example, a previous study from the SNKLR showed that the ACL revision group reported approximately 14 points lower 1-year KOOS sport and recreation compared with a primary ACL reconstruction group [8]. In the present study, the KOOS sports and recreation was only approximately five points lower 1 year after ACL revision compared with primary ACL reconstruction, despite the fact that the score after ACL revision was similar to in the prior SNKLR study [8]. Thus, the smaller difference between primary and revision found in present study is a result of a generally lower KOOS sports and recreation among the patients undergoing subsequent ACL revision, suggesting that patients undergoing an ACL revision are able to attain a fairly similar KOOS after their revision surgery compared with their primary ACL reconstruction.

There was an increase in concomitant cartilage injuries from primary to revision ACL reconstruction, which is in concordance with previous literature [5–7]. More than one in four patients with no cartilage injury at primary surgery had one at ACL revision (233 patients of the 828 patients with no cartilage injury at primary ACL reconstruction), which is a notable proportion when considering that the mean time between the surgeries was less than 3 years. In agreement with data from the MARS cohort [19], severe cartilage injury (ICRS 3-4) was a significant predictor of a poorer KOOS after ACL revision even though the clinical relevance of the effect in the multivariable analysis of the KOOS sports and recreation is questionable. It should however be emphasized that the short follow-up in the present study means that the outcome after ACL revision may still deteriorate over time. In a mid-term perspective (nearly 5 years after ACL revision), a poorer patient-reported outcome and a lower rate of return to pre-injury activity level have been reported for patients with ICRS 3-4 cartilage injuries and medial meniscal injuries [20]. It is possible that such a time perspective is needed to detect clinically relevant effects of concomitant cartilage injuries, which the SNKLR unfortunately not yet permits since the registry was established in 2005 and there is a scarce number of patients with 5-year follow-up for both primary and revision ACL reconstruction. The comparable short-term outcome found between primary and revision ACL reconstruction, despite an increase in cartilage injuries, could still have clinical implications. Knowledge that an ACL revision patient might perceive the knee as fairly well-functioning in the short-term despite severe cartilage injury needs to be considered with regard to future lifestyle choices. Although it may enable a return to knee-strenuous activity shortly after ACL revision, this decision needs to be weighed up against the possible long-term consequences of the continued propagation of intra-articular injuries and post-traumatic osteoarthritis.

The use of allograft for ACL revision was an independent predictor for poorer KOOS with an effect likely to represent a clinically relevant inferiority. Similar results have been reported by the MARS group [21], which also reported a 2.78 times greater risk of graft rupture with the use of allograft compared with autograft [21]. It is obvious that there is a difference in clinical practice between Sweden and the USA with regard to the use of allografts, since only 7% in the current cohort compared with 49% in the MARS cohort [21] underwent allograft ACL revision. Although the present analysis supports the findings from the MARS cohort, it is limited by a small allograft group. Moreover, there is evidence that that the mechanical strength is reduced in irradiated allografts [22–24], and it remains unknown whether the use of irradiated allografts might have contributed to the result since information regarding the sterilization process is not kept in the SNKLR. It should be remembered that allografts are a heterogenous group, including different tissues and sterilization methods, and the lack of high-level studies assessing the outcome with allografts in ACL revision limits the ability to draw definite conclusions on this topic [25–27]. It is also likely that an inferior outcome with allograft is not a direct consequence of the graft itself but is closely related to the reasons for selecting an allograft for ACL revision—such as surgeon preferences, concomitant knee injuries, multi-ligament reconstruction, postoperative desired patient activity level and expectations, plus patient demographics [28].

A PLC injury at the time of revision had a detrimental effect on the 1-year KOOS after ACL revision, predicting a 25- to 30-point lower KOOS_4_, sports and recreation, and QoL. Although there were few PLC injuries in this cohort overall, the prevalence of a PLC injury increased four-fold from primary to ACL revision. The complex anatomy of the PLC, in combination with the challenging physical examination in the acute setting of multiple ligament injuries, means that injuries to the PLC may be misdiagnosed [29–31]. Although the present study is unable to prove any causality between the increased prevalence of PLC injuries at ACL revision, it is not unlikely that failure to detect a PLC injury at primary ACL reconstruction may have contributed to the ACL failure in some patients [32]. The treatment of the PLC injuries at the time of ACL revision in this study was not investigated and, although recent studies have reported fair clinical outcomes following the surgical reconstruction of the PLC in combination with ACL reconstruction in the primary surgery [33, 34], less is known about the outcome of such a procedure with an ACL revision. It must be stated that the reasons for the impairment predicted by a PLC injury in the present study remain unknown and the findings should be interpreted with care, due to the small number of patients with a PLC injury. Nonetheless, it justifies further investigation and underscores the fact that clinicians need to be highly suspicious of associated PLC injuries when diagnosing an ACL rupture.

There are some limiting factors that should be considered when interpreting the results of this study. The univariable and multivariable analyses explained only a small proportion of the variance in the KOOS. It is possible that a larger study population would have enabled a more robust analysis. It is also possible that the predictors or the various KOOS subscales included in the present study are insufficient to determine which factors are the most important for outcome after ACL revision. Factors such as quality of rehabilitation, knee joint laxity, and functional performance tests most definitely impact the outcome after ACL revision and future studies also including these data are warranted. A robust predictive analysis also requires a relevant patient- and injury-specific outcome measurement that captures important aspects of outcome. It is possible that the KOOS may not be sensitive enough to discriminate between clinically important differences for young patients undergoing ACL reconstruction. Moreover, although all the included patients had KOOS data available for at least one occasion (preoperatively or 1-year) at both the primary and revision ACL reconstruction, the reporting occasions were frequently found not to correspond between the primary and revision ACL reconstruction. Consequently, the study samples for each comparative analysis of the same patient’s KOOS preoperatively and 1 year after primary and revision ACL reconstruction were smaller than the total study cohort. The fact that multiple surgeons report to the SNKLR means that there is a risk of reporting bias for the investigated variables, as well as different subjective assessments and treatments for the intra-articular injuries.

## Conclusions

This study showed that patients undergoing ACL revision reported a 1-year outcome that was slightly inferior to the 1-year outcome after their primary ACL reconstruction. An ACL revision was associated with an increase in cartilage injuries. A PLC injury at ACL revision and the use of allograft for ACL revision predicted a clinically relevant inferior KOOS 1 year after ACL revision.

## Data Availability

The datasets used and/or analyzed during the current study are available from the corresponding authors on reasonable request.

## Appendix

**Table 6:** Univariable analysis for the Knee Osteoarthritis Outcome Score at one-year follow-up after ACL revision with independent variables from the primary ACL reconstruction

		KOOS sport and recreation			KOOS QoL			KOOS_4_		
		Univariable			Univariable			Univariable		
	Value	Mean (SD) Median *n*=	Beta (95% CI)	*p* value	Mean (SD) Median *n*=	Beta (95% CI)	*p* value	Mean (SD) Median *n*=	Beta (95% CI)	*p* value
Any meniscal injury	No	52.11 (30.19) 50.00 *n* = 256			46.44 (25.40) 43.75 *n* = 256			62.18 (21.80) 64.35 *n* = 256		
	Yes	51.57 (30.46) 55.00 *n* = 226	− 0.54 (− 5.98;4.90)	0.85	44.99 (27.04) 43.75 *n* = 226	− 1.44 (− 6.14; 3.25)	0.55	61.25 (23.09) 65.05 *n* = 226	− 0.93 (− 4.95; 3.09)	0.65
MM injury	No	53.17 (29.95) 55.00 *n* = 367			46.32 (25.73) 43.75 *n* = 367			62.47 (22.05) 64.77 *n* = 367		
	Yes	47.65 (31.11) 50.00 *n* = 115	− 5.52 (− 11.87; 0.83)	0.088	43.97 (27.57) 43.75 *n* = 115	− 2.35 (− 7.85; 3.14)	0.40	59.44 (23.40) 62.77 *n* = 115	− 3.03 (− 7.72; 1.67)	0.21
LM injury	No	51.79 (30.46) 50.00 *n* = 330			46.42 (26.01) 43.75 *n* = 330			62.16 (22.07) 64.88 *n* = 330		
	Yes	52.01 (30.02) 55.00 *n* = 152	0.22 (− 5.62; 6.06)	0.94	44.33 (26.54) 43.75 *n* = 152	− 2.09 (− 7.14; 2.95)	0.41	60.85 (23.12) 63.99 *n* = 152	− 1.31 (− 5.63; 3.00)	0.55
Meniscal Surgical treatment	No	52.29 (30.49) 50.00 *n* = 286			46.35 (25.72) 43.75 *n* = 286			62.18 (22.26) 64.65 *n* = 286		
	Yes	51.22 (30.06) 55.00 *n* = 196	− 1.07 (− 6.59; 4.46)	0.70	44.90 (26.85) 43.75 *n* = 196	− 1.45 (− 6.22; 3.32)	0.55	61.11 (22.63) 64.63 *n* = 196	− 1.07 (− 5.15; 3.01)	0.61
Meniscal resection	No	52.20 (30.43) 50.00 *n* = 316			45.95 (25.88) 43.75 *n* = 316			62.00 (22.37) 64.46 *n* = 316		
	Yes	51.20 (30.11) 55.00 *n* = 166	− 0.99 (− 6.70; 4.72)	0.73	45.41 (26.78) 43.75 *n* = 166	− 0.54 (− 5.47; 4.39)	0.83	61.26 (22.50) 65.05 *n* = 166	− 0.75 (− 4.97; 3.48)	0.73
Meniscal repair	No	51.53 (30.41) 50.00 *n* = 444			45.89 (26.20) 43.75 *n* = 444			61.64 (22.42) 64.49 *n* = 444		
	Yes	55.66 (28.97) 60.00 *n* = 38	4.13 (− 5.94; 14.19)	0.42	44.24 (26.09) 37.50 *n* = 38	− 1.65 (− 10.34; 7.05)	0.71	62.97 (22.35) 66.23 *n* = 38	1.33 (− 6.11; 8.78)	0.73
Cartilage injury	No	51.99 (29.60) 55.00 *n* = 395			45.73 (25.44) 43.75 *n* = 395			62.07 (21.54) 64.55 *n* = 395		
	Yes	51.26 (33.40) 50.00 *n* = 87	− 0.72 (− 7.78;6.33)	0.84	45.91 (29.41) 43.75 *n* = 87	0.18 (− 5.92; 6.27)	0.95	60.27 (26.00) 66.57 *n* = 87	− 1.80 (− 7.01; 3.42)	0.50
Highest grade on any cartilage injury	No injury	51.99 (29.60) 55.00 *n* = 395			45.73 (25.44) 43.75 *n* = 395			62.07 (21.54) 64.55 *n* = 395		
	ICRS 1-2	51.64 (33.91) 60.00 *n* = 70			46.61 (29.94) 46.88 *n* = 70			60.60 (26.30) 69.14 *n* = 70		
	ICRS 3-4	49.71 (32.18) 50.00 *n* = 17	− 0.76 (− 6.30; 4.78)	0.79	43.01 (27.77) 37.50 *n* = 17	− 0.29 (− 5.08; 4.49)	0.90	58.94 (25.43) 52.72 *n* = 17	− 1.52 (− 5.62; 2.57)	0.47
Cartilage injury patella	ICRS 0-2	51.76 (30.24) 50.00 *n* = 481			45.65 (26.08) 43.75 *n* = 481			61.67 (22.35) 64.56 *n* = 481		
	ICRS 3-4	100.00 (.) 100.00 *n* = 1	48.24 (− 11.24; 107.73)	0.11	100.00 (.) 100.00 *n* = 1	54.35 (3.06; 105.64)	0.038	100.00 (.) 100.00 *n* = 1	38.33 (− 5.63; 82.29)	0.087
Cartilage injury tibial plateau	ICRS 0-2	52.04 (30.23) 55.00 *n* = 478			45.95 (26.16) 43.75 *n* = 478			61.90 (22.31) 64.73 *n* = 478		
	ICRS 3-4	30.00 (33.42) 25.00 *n* = 4	− 22.04 (− 51.89; 7.81)	0.15	23.44 (16.44) 28.13 *n* = 4	− 22.51 (− 48.27; 3.25)	0.087	43.10 (28.40) 46.39 *n* = 4	− 18.81 (− 40.86; 3.25)	0.094
Cartilage injury femoral condyles	ICRS 0-2	51.87 (30.39) 55.00 *n* = 469			45.80 (26.25) 43.75 *n* = 469			61.79 (22.45) 64.77 *n* = 469		
	ICRS 3− 4	51.54 (27.57) 50.00 *n* = 13	− 0.33 (− 17.08; 16.42)	0.97	44.23 (23.73) 37.50 *n* = 13	− 1.57 (− 16.04;12.90)	0.83	60.15 (20.94) 52.72 *n* = 13	− 1.65 (− 14.03; 10.74)	0.79
Cartilage injury trochlea	ICRS 0− 2	51.86 (30.29) 52.50 *n* = 482	0.00 (.;.)	.	45.76 (26.17 ) 43.75 *n* = 482	0.00 (.;.)	.	61.75 (22.39) 64.63 *n* = 482	0.00 (.;.)	.
	ICRS 3-4	*n* = 0			*n* = 0			*n* = 0		
MCL injury	No	52.12 (30.40) 55.00 *n* = 460			45.95 (26.37) 43.75 *n* = 460			61.95 (22.48) 64.76 *n* = 460		
	Yes	46.36 (28.04) 50.00 *n* = 22	− 5.76 (− 18.75;7.24)	0.38	41.76 (21.60) 40.63 *n* = 22	− 4.19 (− 15.42;7.04)	0.46	57.57 (20.50) 59.60 *n* = 22	− 4.38 (− 13.98;5.23)	0.37
LCL injury	No	51.96 (30.31) 52.50 *n* = 478			45.80 (26.19) 43.75 *n* = 478			61.83 (22.37)64.63 *n* = 478		
	Yes	40.00 (29.72) 47.50 *n* = 4	− 11.96 (− 41.85;17.94)	0.43	40.63 (26.27) 43.75 *n* = 4	− 5.18 (− 31.02; 20.66)	0.69	51.53 (26.16) 56.89 *n* = 4	− 10.30 (− 32.40; 11.79)	0.36
PCL injury	No	51.98 (30.15) 55.00 *n* = 475			45.87 (26.05) 43.75 *n* = 475			61.89 (22.20) 64.70 *n* = 475		
	Yes	43.57 (41.00) 45.00 *n* = 7	− 8.41 (− 31.08; 14.26)	0.47	38.39 (34.69) 18.75 *n* = 7	− 7.48 (− 27.06; 12.11)	0.45	52.34 (34.11) 48.18 *n* = 7	− 9.55 (− 26.30; 7.20)	0.26
PLC injury	No	52.00 (30.25) 55.00 *n* = 480			45.90 (26.13) 43.75 *n* = 480			61.87 (22.34) 64.73 *n* = 480		
	Yes	17.50 (24.75) 17.50 *n* = 2	− 34.50 (− 76.60; 7.60)	0.11	12.50 (8.84) 12.50 *n* = 2	− 33.40 (− 69.75; 2.95)	0.072	32.90 (23.41) 32.90 *n* = 2	− 28.97 (− 60.07; 2.13)	0.068

*ACL* anterior cruciate ligament, *ICRS* international cartilage repair score, *MCL* medial collateral ligament, *MM* medial meniscus, *LCL* lateral collateral ligament, *LM* lateral meniscus, *PCL* posterior cruciate ligament, *PLC* posterolateral corner

## Abbreviations

ACL: Anterior cruciate ligament
ADL: Activities of daily living
CI: Confidence interval
EQ-5D: European Quality of Life-5 Dimensions
ICRS: International cartilage repair society
KOOS: Knee Injury and Osteoarthritis Outcome Score
LCL: Lateral collateral ligament
MCL: Medial collateral ligament
PLC: Posterolateral corner
QoL: Quality of life
SD: Standard deviation
SNKLR: Swedish national knee ligament registry
